# Pharmacokinetics after Periocular Methylprednisolone Sodium Succinate Injection in Rabbit Eyes

**DOI:** 10.1155/2020/8120398

**Published:** 2020-02-27

**Authors:** Hua-Yi Lu, Rui-Qing Wang, Xin Li, Xu Li

**Affiliations:** ^1^Department of Ophthalmology, The Second Hospital of Jilin University, Changchun, Jilin Province 130021, China; ^2^The First Affiliated Hospital of USTC, Division of Life Sciences and Medicine, University of Science and Technology of China, Hefei, Anhui 230001, China

## Abstract

The present study aimed to determine the pharmacokinetics and distribution of methylprednisolone sodium succinate (MPSS) and its metabolic product methylprednisolone (MP) in plasma and ocular tissues after periocular injection of MPSS in rabbit eyes. Forty-eight healthy New Zealand white rabbits were randomly divided into 12 groups, including the control group and 11 MPSS-treated groups sampling at different time points. Rabbits in the MPSS-treated groups underwent left eye periocular injection of MPSS (10 mg). The pharmacokinetics of MPSS and MP in plasma and ocular tissues (including aqueous humor, vitreous, iris, lens, sclera, optic nerve, and choroid and retina) were investigated by liquid chromatography tandem mass spectrometry (LC-MS/MS). After periocular injection, the time of maximum concentration (*T*_max_) of MPSS ranged from 0.25 h to 1 h in ocular tissues and was 0.25 h in plasma. *T*_max_ of MP in ocular tissues ranged from 0.5 h to 6 h, and *T*_max_ of MP in plasma was 0.5 h. The maximum concentration (*C*_max_) of MPSS and MP and the area under the curve (AUC_0-*t*_) in ocular tissues from high to low was sclera, optic nerve, choroid and retina, iris, and lens. Especially, the concentrations of MPSS and MP in the lens were much lower when compared with the other ocular tissues. After periocular administration, MPSS could be rapidly metabolized to its active constituent MP in the ocular tissues. Also, the MPSS can be delivered effectively into the posterior segment of the eye (choroid and retina), while not easily be absorbed by the lens.

## 1. Introduction

Corticosteroids have been the mainstay in the treatment of ocular inflammatory disorders for more than 60 years [1]. Corticosteroids could be administrated topically (eyedrop and intravitreal or periocular injection) or systemically (orally or intravenous injection) [2]. Among topical treatments to eye infection, eyedrop is the least invasive method, but the drugs only penetrate well into the anterior segment of the eye. Thus, eyedrop is not applicable for patients with posterior segment lesions. Systemically administered corticosteroids have to cross the blood-retinal barrier (BRB), a selective partition between the retina and circulation. Therefore, systemic administration is not an effective method of delivering drugs to the vitreous and retina. Moreover, the long-term use of systemic corticosteroids will bring many side effects, such as femoral head necrosis, central obesity, infections, and peptic ulcers. The recently developed intravitreal injection can bypass the BRB, allowing the drugs to reach high concentrations in the vitreous and retina. However, intravitreal administration is the most invasive delivery route and causes serious and high-frequency complications, such as ocular hypertension and cataracts, making intravitreal injection a controversial route. Hence, many ophthalmologists focus on periocular injection again. Periocular administration can help achieve sufficient and high topical drug concentration, prolong the duration of drug action, limit the systemic exposure, and decrease the occurrence of adverse events. Periocular injection can be conducted repeatedly when needed. Howbeit, the challenge of periocular injection is that the drug penetration from the periocular space to the retina must cross multiple obstacles, including the sclera, vascularized choroid, and retinal pigment epithelium.

Due to the difficulty in intraocular penetration, drugs with strong efficacy and long-lasting effects are favorable. Methylprednisolone sodium succinate (MPSS; C_26_H_33_NaO_8_, molecular weight, 496.53) is an artificial synthetic glucocorticoid with the characteristics of rapid response, potent efficacy, and minimal side effects. Its active pharmaceutical ingredient is methylprednisolone. The pharmacological effect of MPSS is 5 times that of hydrocortisone. Compared with other corticosteroids, such as prednisolone, the pharmacokinetic properties of MPSS are simple, showing first-order kinetics. There is no obvious dose or time dependence. MPSS has been commonly used in ocular inflammatory diseases by oral administration or periocular or intravenous injection [3, 4]. The pharmacokinetics of MPSS after oral and intravenous administration in plasma had been revealed by high performance thin layer and high pressure liquid chromatography [5]. However, there are few studies about the pharmacokinetics of MPSS and its metabolic product methylprednisolone (MP) after periocular injection. Thus, it might be of great importance in clinical evaluation of MPSS to uncover the pharmacokinetics of MPSS and MP after periocular injection. In the present study, we investigated the pharmacokinetics and distribution of MPSS and MP in plasma and ocular tissues (including the aqueous humor, vitreous, iris, lens, sclera, optic nerve, and choroid and retina) of rabbits after periocular injection.

## 2. Materials and Methods

### 2.1. Animals, Treatment, and Sampling

In the present study, 48 healthy New Zealand white rabbits without any eye disease (24 males and 24 females; weighting 2.0–2.5 kg) were purchased from Tianjin Medical Laboratory Animal Center. Animal care and use was conducted in compliance with the Regulations for the Administration of Affairs Concerning Experimental Animals of China (http://www.gov.cn/gongbao/content/2014/content_2692743.htm). The experiment was approved by the Ethics Committee of Second Hospital of Jilin University in China.

The above healthy 48 rabbits were randomly divided to 12 groups, including the control group (*n* = 4) and 11 MPSS-treated groups (*n* = 4 per group) with male and female in half. In the control group, rabbits received no treatment and were sacrificed by ear marginal venous air embolism after anesthesia to collect samples (considered as 0 hour). Rabbits in the 11 MPSS-treated groups were given left eye periocular injection of 10 mg MPSS (Pfizer Manufacturing Belgium NV, Puurs, Belgium). Rabbits in the 11 MPSS-treated groups were sacrificed to obtain samples at 0.25, 0.5, 1, 1.5, 2, 3, 4, 6, 12, 24, and 48 hours after MPSS injection, respectively.

The venous blood, aqueous humor, vitreous, iris, lens, sclera, optic nerve, and choroid and retina samples were collected from all the four rabbits in each group by the researchers from the Research Center for Drug Metabolism of Jilin University. Auricular venous blood (1500 *μ*L) was collected for isolation of plasma by centrifugation (15000 rpm for 10 min), and then isolated plasma samples were stored at −20°C for use. The eyes were enucleated, rinsed with cold physiological saline, wiped dry, and dissected under a microscope. Aqueous humor (≥300 *μ*L) was aspirated and then stored at −20°C. The vitreous humor was aspirated and centrifuged at 3500 rpm for 5 min, and the supernatant was stored at −20°C. The other isolated ocular samples including the iris, lens, sclera, optic nerve (5 mm from the optic disc), and choroid and retina were put in a 10 ml EP tube, and 1 ml of cold methanol-water (1 : 1, v/v) was added to dissolve the samples. Then, the EP tube was placed in ice water, and homogenate was conducted with a homogenizer for 30 sec. Another 1 ml of cold methanol-water (50 : 50, v/v) was added in each tube. The mixture was sonicated for 30 sec and then centrifuged at 10,000 rpm for 10 min at 4°C. The supernatant was stored at −20°C for use.

### 2.2. Pharmacokinetic Analysis

#### 2.2.1. Sample Preparation

Samples (200 *μ*L plasma, aqueous humor, vitreous, 500 *μ*L of iris, lens, sclera, optic nerve, and choroid and retina), the internal standard solution (100 *μ*L), and methanol-water (50 : 50, v/v; 200 mL) were mixed together, respectively. Then, 3 ml ether/dichloromethane mixture (2 : 1, v/v) was added. The mixture was vortexed for 1 min, shaken for 10 min (240 beats/min), and then centrifuged for 5 min at 3500 rpm. The supernatant was collected into another tube and dried under a stream of nitrogen at 40°C. Afterwards, 100 *μ*L mobile phase containing acetonitrile and ammonium acetate (50 : 50, v/v; pH 5.0) was added into supernatant, and the mixture was vortexed.

#### 2.2.2. Instrumentation and Conditions

Four samples of plasma and ocular tissues (including aqueous humor, vitreous, iris, lens, sclera, optic nerve, and choroid and retina) were measured by LC-MS/MS for each group, including control group (0 h) and 11 MPSS-treated groups (sampling at 0.25, 0.5, 1, 1.5, 2, 3, 4, 6, 12, 24, and 48 h after MPSS injection). According to the methods reported by previous studies [6–8], 40 *μ*L prepared samples were used for liquid chromatography tandem mass spectrometry (LC-MS/MS) consisted of an Agilent 1100 series HPLC (Agilent Technologies, Palo Alto, CA, USA) coupled to an Applied Biosystems Sciex Q-trap™ mass spectrometer (Applied Biosystems Sciex, Ontario, Canada). Chromatography was performed on a Venusil Mp-C18 column (4.6 × 50 mm ID, 5 *μ*m particle size; Agela Technologies Inc., USA) at 40°C using a mobile phase constituted with acetonitrile-1 mM ammonium acetate solution (50 : 50, v/v; pH 5.0). The flow rate was 1.0 mL/min. The column effluent was split into the detector, which was equipped with an ion-spray ionization source and operated in the negative ion mode. The parameters were as follows: ion-spray voltage, −4500 V; temperature, 550°C; source gas 1 (GS1, N2) pressure, 75 psi; gas 2 (GS2, N2) pressure, 75 psi; and gas curtain gas (N2) pressure, 10 psi. Multiple reaction monitoring (MRM) was conducted. The declustering voltages (DVs) of MP, MPSS, and dexamethasone control were −115 V, −88 V, and −15 V, and the collision energy (CE) was −14 eV, −25 eV, and −25 eV, respectively. Data acquisition was conducted by Analyst 1.3 software.

### 2.3. Statistical Analysis

All results are expressed as the mean ± standard deviation (SD). The maximum concentration (*C*_max_) and time to maximum concentration (*T*_max_) were recorded as observed from the concentration-time curve. Area under the concentration-time curve from time 0 to the time of the last point (AUC_0-*t*_) was calculated by the trapezoidal rule. The elimination rate constant (*k*) was calculated by log-linear regression of concentration during the elimination phase, and elimination half-life (*t*_1/2_) was calculated by 0.693/*k*. Area under the curve to time infinity (AUC_0-∞_) was calculated by adding AUC_0-*t*_ with Clast/*k*, where Clast was the last quantifiable concentration. The area-under-the-moment-curve (AUMC) was calculated by the method of statistical moments. The mean residence time (MRT) was calculated as the AUMC/AUC, where AUC is the area under the concentration curve from time 0 to infinity.

## 3. Results

### 3.1. Mass Spectrometry

**Figure 1:**
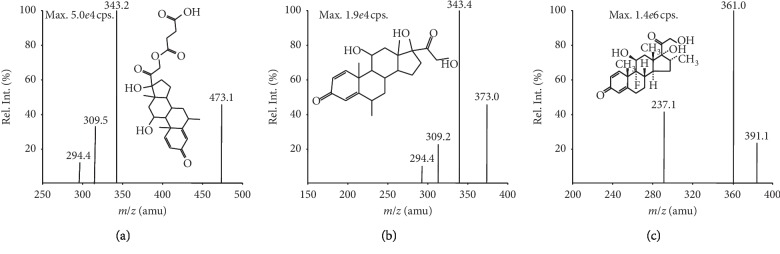
Full scan product ion spectra of [M-H]^−^ for MPSS (a), MP (b), and dexamethasone (c).

**Figure 2:**
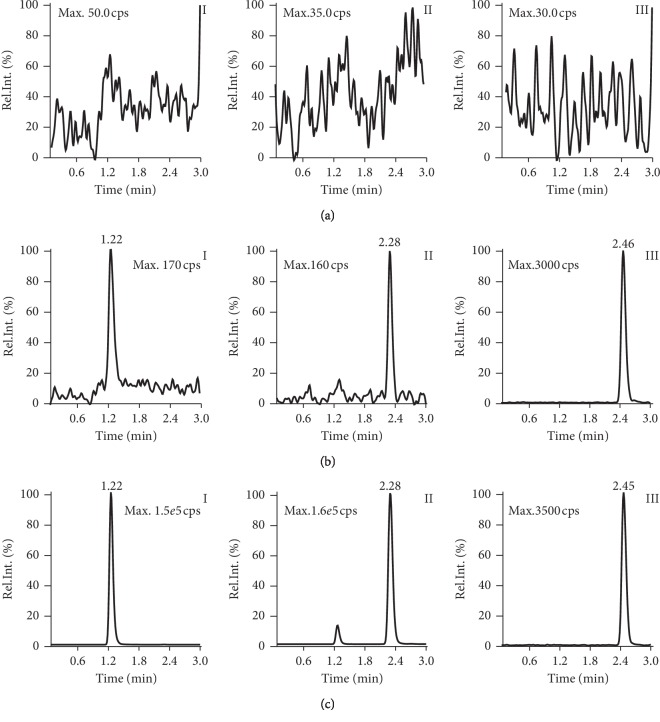
Representative multiple reaction monitoring chromatograms of MPSS, MP, and dexamethasone in rabbit blood plasma: (a) blank plasma sample; (b) plasma sample spiked with MPSS (0.3 ng/ml), MP (3 ng/ml), and dexamethasone (1000 ng/ml); (c) plasma sample obtained when concentration was highest after administration of MPSS in healthy rabbits (I for MPSS, II for MP, and Ш for dexamethasone).

MRM was performed using the mass transition ion-pairs *m*/*z* 473.1 ⟶ *m*/*z* 343.2 for MPSS, *m*/*z* 373 ⟶ *m*/*z* 343.4 for MP, and *m*/*z* 391.1 ⟶ *m*/*z* 361.0 for dexamethasone (Figure 1). Typical chromatograms are shown in Figure 2.

### 3.2. Pharmacokinetic Data

After periocular injection of MPSS, MPSS and its metabolite (MP) could be detected in all of the collected samples. The concentration-time profile of MPSS is shown in Table 1. The highest *C*_max_ was observed in plasma (4478.0 ± 437.4 ng/mL), followed by the vitreous (3271.0 ± 332.4 ng/mL) and aqueous humor (822.0 ± 114.7 ng/mL). The highest *C*_max_ of MPSS in eye tissues was detected in the sclera (48050.0 ± 3223.8 ng/mL), followed by the optic nerve (1627.0 ± 212.9 ng/mL), choroid and retina (1052.0 ± 104.6 ng/mL), iris (776.0 ± 68.7 ng/mL), and lens (252.0 ± 28.9 ng/mL). The concentration-time profile of MP is shown in Table 2. The highest *C*_max_ of MP was observed in plasma (1290.0 ± 269.0 ng/mL), followed by the aqueous humor (643.0 ± 93.0 ng/mL) and vitreous (329.0 ± 67.0 ng/mL). The highest *C*_max_ in eye tissues was detected in the sclera (3960.0 ± 820.0 ng/mL), followed by the optic nerve (542.0 ± 67.0 ng/mL), choroid and retina (504.0 ± 54.0 ng/mL), iris (254.0 ± 54.0 ng/mL), and lens (22.0 ± 5.6 ng/mL).

*T *_max_ of MPSS was 0.25 h in the plasma, iris, sclera, and choroid and retina, 0.5 h in the vitreous, lens, and optic nerve, and 1 h in the aqueous humor (Table 3). *T*_max_ of MP was 0.5 h in the plasma, iris, sclera, optic nerve, and choroid and retina, 4 h in the aqueous humor, and 6 h in the lens. In plasma, *t*_1/2_ of MPSS and MP was 13.2 h and 3.29 h after injection of MPSS, and the MRT of MPSS and MP was 0.826 h and 2.78 h, respectively. The MRT of MPSS (12.2 h) and MP (16.1 h) was the longest in the lens, whereas the maximum *k* of MPSS (36.5% per h) and MP (38.9% per h) was found in the aqueous humor. Sclera had the maximum AUC of MPSS, followed by the vitreous, aqueous humor, plasma, optic nerve, choroid and retina, iris, and lens.

## 4. Discussion

The major problems of corticosteroid application in ocular diseases are the poor penetration to posterior segment and the systemic adverse events, and the goal of the drug delivery system is to deliver the drug at the right target sites with appropriate concentrations. The aim of this study was to assess whether MPSS could rapidly penetrate into the eye tissues and fluids after periocular injection.

We found that the concentrations of MPSS in the eye tissues peaked at 0.25–1 h after injection and then decreased rapidly. This indicated that MPSS can be quickly absorbed by intraocular tissues after periocular injection, which is the premise of the rapid drug effect. In addition to the aqueous humor and lens, peak times of MP in ocular tissues and plasma were 0.5 h after injection, suggesting that MPSS was quickly metabolized into its active form. The late peak time for MP in the aqueous humor and lens may be caused by the penetrating process of drugs (including MPSS and its metabolite) from the adjacent tissue.

This study also showed that the MRT of MPSS and MP in plasma was 0.826 h and 2.78 h, respectively, indicating that the effect of MPSS and its product MP on systemic tissues was transient and could be eliminated rapidly. Therefore, topical periocular application of MPSS can effectively avoid the systemic side effects. This route of MPSS administration might be particularly suitable for patients complicated by systemic system diseases. Among the collected ocular tissues and fluids, MPSS and MP in the lens had the longest MRT, which might result from the slow metabolism and lacking of enzymes that convert MP or MPSS in the lens. However, this phenomenon may reduce the incidence of corticosteroid-induced cataracts.

After periocular injection, the highest peak concentration of MPSS in the intraocular tissues was detected in the sclera, followed by the optic nerve, choroid and retina, iris, and lens. As to fluids, the highest peak concentration was observed in the plasma, followed by vitreous. *C*_max_ of plasma and vitreous were much higher than that of the aqueous humor. Maximum AUC was shown in the sclera, followed by the vitreous, aqueous humor and plasma, showing easier accumulation of MPSS in eye tissues than in plasma. In addition, majority of MPSS reached the posterior segment, suggesting that periocular injection delivered MPSS was suitable for posterior segment lesions. The concentrations of MP were higher in the plasma, aqueous humor, and vitreous. In vitreous, MPSS exhibited a much higher level than MP. The vitreous is consisted of 99% of water and 1% of collagen, thus lacking enzymes that hydrolyze MPSS. Also, MPSS may have a stronger affinity with intraocular tissues, and its metabolic rates in the iris, aqueous humor, and choroid and retina were much higher than that in the vitreous. The previous pharmacokinetic study showed that, after intravenous injection of 500 mg MPSS in human eye, the drug concentration in vitreous was 1/10 of that in serum [9]. The administration route of periocular injection greatly reduced the dosage of drugs, decreased the incidence of systemic side effects, and significantly improved the bioavailability of drugs. As a commonly used peribulbar-injected drug for the posterior segment eye diseases, triamcinolone acetonide (TA) had a *T*_max_ of 1 d in the vitreous and a MRT of 16 d after injecting into the posterior subtenon of vitrectomized rabbit eyes [10]. Compared with TA, MPSS had shorter *T*_max_ (0.5 h) and MRT (4.44 h) in vitreous after peribulbar administration, indicating a rapid effect and short residence time.

In the pharmacokinetic studies of eye tissues, the intravitreal concentration of drugs is often selected as the evaluation basis, due to the limitation of intraocular materials acquisition. The present study suggested that it was not possible to determine the amount of drugs entering the eye or whether it can be absorbed or utilized by eye tissues based solely on the intravitreal drug concentration. In fact, the effects of corticosteroid application are mainly about reducing edema, exudation, and other inflammatory reactions of the choroid and retina. Since drugs mainly play effects in the choroid and retina, more attention should be paid on the distribution of drugs in the choroid and retina. In this study, the largest AUC of MPSS was found in the sclera, followed by the vitreous, aqueous humor, plasma, optic nerve, choroid and retina, iris, and lens. With the exception of the sclera and optic nerve that might directly absorb the MPSS, the MPSS exposure in the choroid and retina was higher than that in the iris and much higher than that in the lens, suggesting that periocular injection was an effective way to deliver MPSS to the posterior segment of the eyes. *T*_max_ of MPSS was 0.25–1 h, indicating that the intraocular drug concentrations could get up to the highest quickly. The MRT of MPSS ranged from 0.826 h to 12.2 h, and the choroid and retina showed an intermediate level of 8.71 h.

The MRT in the sclera was relatively short, which might be due to that the drug firstly reached the sclera after periocular injection, resulting in the highest drug concentration and AUC in the sclera. Subsequently, the drug may be transferred into blood and circulate into ocular tissues and other parts of the body, be metabolized, or penetrated through the sclera to the intraocular tissues. The sclera has a large surface area and a high degree of hydrophilicity, which makes water-soluble macromolecular drugs (relative molecular mass of 70,000 to 150,000) to penetrate into intraocular tissues. In addition, there are few cells in the sclera, and the sclera lacks proteolytic enzymes; thus, some macromolecular biological active substances (such as oligonucleotides and monoclonal antibodies) can reach to the choroid and retina through the sclera [11–14]. In short, after periocular injection of MPSS, the sclera is the main barrier for drug penetration, but the sclera also is a “repository” for local drug release due to its large surface area.

There are many administration routes to deliver drugs into ocular tissues. The choice is primarily made based on the desired target site for the drug. Conventional ocular topical administration and subconjunctival administration are mainly used for the anterior segment lesions, whereas intravitreal administration and transscleral administration are for the posterior segment lesions. This study provides a theoretical basis for the transscleral administration of some new pharmaceutical dosage forms, such as the hormone scleral delivery system [15].

## 5. Conclusion

The present study aimed to study the pharmacokinetics of MPSS after periocular injection in rabbit eyes. MPSS was rapidly absorbed in the intraocular tissues, effectively delivered to the posterior segment of the eyes (choroid and retina) and then quickly eliminated. The prodrug could rapidly convert into its active ingredient (MP) in eye tissues. Hence, periocular injection is an effective way to deliver drugs to the eyes.

## Figures and Tables

**Table 1:** Concentrations (ng/mL) of MPSS in tissues and plasma at different time points in 44 rabbits after periocular injection.

Time points (h)	Plasma	Vitreous	Aqueous humor	Iris	Sclera	Lens	Choroid and retina	Optic nerve
0	0.0 ± 0.0	0.0 ± 0.0	0.0 ± 0.0	0.0 ± 0.0	0.0 ± 0.0	0.0 ± 0.0	0.0 ± 0.0	0.0 ± 0.0
0.25	4478.0 ± 437.4	1987.0 ± 254.9	563.0 ± 54.4	776.0 ± 68.7	48050.0 ± 3223.8	190.0 ± 30.1	1052.0 ± 104.6	1078.0 ± 120.7
0.5	910.0 ± 68.7	3271.0 ± 332.4	145.0 ± 13.3	348.0 ± 34.1	38752.0 ± 4181.7	252.0 ± 28.9	537.0 ± 47.3	1627.0 ± 212.9
1	335.0 ± 16.8	289.0 ± 21.3	822.0 ± 114.7	179.0 ± 16.2	24345.0 ± 2650.8	34.1 ± 5.2	201.0 ± 23.7	877.0 ± 124.2
1.5	210.0 ± 11.5	582.0 ± 46.7	546.0 ± 73.9	43.6 ± 3.1	22595.0 ± 1485.90	13.4 ± 2.2	445.0 ± 40.5	179.0 ± 27.4
2	191.0 ± 14.3	656.0 ± 61.2	271.0 ± 31.2	156.0 ± 15.0	16525.0 ± 1584.6	7.7 ± 1.7	584.0 ± 53.7	343.0 ± 41.2
3	26.6 ± 4.5	139.0 ± 15.6	280.0 ± 23.8	13.9 ± 1.9	2455.0 ± 301.9	2.5 ± 0.7	66.0 ± 8.5	22.2 ± 2.7
4	4.2 ± 0.6	64.3 ± 18.3	438.0 ± 27.4	17.8 ± 1.4	1024.0 ± 145.6	6.0 ± 0.8	26.4 ± 3.4	48.9 ± 6.1
6	2.4 ± 0.7	104.0 ± 14.1	208.0 ± 17.0	10.3 ± 1.1	464.0 ± 30.7	15.9 ± 1.4	9.0 ± 1.3	4.0 ± 0.5
12	0.8 ± 0.1	3.2 ± 1.4	10.7 ± 11.5	4.4 ± 0.7	37.1 ± 2.8	2.3 ± 0.5	1.8 ± 0.5	2.0 ± 0.2
24	0.4 ± 0.1	3.4 ± 1.6	0.3 ± 0.1	5.3 ± 0.5	53.4 ± 4.3	7.7 ± 0.4	23.6 ± 3.6	3.0 ± 0.8
48	0.2 ± 0.1	14.6 ± 5.3	0.0 ± 0.0	4.6 ± 0.7	165.0 ± 23.7	2.0 ± 0.3	7.0 ± 1.6	5.4 ± 0.7

MPSS, methylprednisolone sodium succinate.

**Table 2:** Average concentrations (ng/mL) of MP in tissues and plasma at different time points in 44 rabbits after periocular injection.

Time points (h)	Plasma	Vitreous	Aqueous humor	Iris	Sclera	Lens	Choroid and retina	Optic nerve
0	0.0 ± 0.0	0.0 ± 0.0	0.0 ± 0.0	0.0 ± 0.0	0.0 ± 0.0	0.0 ± 0.0	0.0 ± 0.0	0.0 ± 0.0
0.25	983.0 ± 143.0	123.0 ± 44.0	187.0 ± 60.0	106.0 ± 36.9	2760.0 ± 728.0	15.4 ± 3.3	163.0 ± 32.0	109.0 ± 76.0
0.5	1290.0 ± 269.0	329.0 ± 67.0	119.0 ± 41.4	254.0 ± 54.0	3960.0 ± 820.0	21.6 ± 4.3	504.0 ± 54.0	542.0 ± 67.0
1	1070.0 ± 189.0	73.3 ± 29.3	365.0 ± 35.8	170.0 ± 54.0	3381.0 ± 874.0	7.8 ± 1.2	343.0 ± 78.0	261.0 ± 19.0
1.5	1040.0 ± 178.0	134.0 ± 47.2	643.0 ± 93.0	40.6 ± 6.0	2612.0 ± 784.0	13.3 ± 2.7	235.0 ± 65.0	165.0 ± 12.0
2	782.0 ± 92.4	201.0 ± 63.0	380.0 ± 77.0	59.1 ± 8.1	2578.0 ± 715.0	8.9 ± 1.7	229.0 ± 43.0	59.9 ± 8.5
3	320.0 ± 69.0	78.4 ± 21.2	358.0 ± 75.0	12.8 ± 1.9	412.0 ± 97.0	5.8 ± 0.8	35.6 ± 5.2	29.1 ± 1.6
4	263.0 ± 58.0	92.4 ± 34.2	683.0 ± 91.0	33.8 ± 4.4	658.0 ± 79.0	11.2 ± 3.3	37.4 ± 7.6	35.6 ± 3.0
6	123.0 ± 49.6	42.3 ± 14.3	399.0 ± 65.0	15.7 ± 3.3	227.0 ± 29.0	22.0 ± 5.6	17.3 ± 1.9	7.1 ± 1.6
12	14.6 ± 6.8	5.5 ± 1.1	17.1 ± 5.4	1.9 ± 0.2	1.7 ± 0.3	8.5 ± 1.1	1.0 ± 0.2	0.0 ± 0.0
24	3.5 ± 2.4	0.7 ± 0.4	0.0 ± 0.0	1.5 ± 0.4	8.1 ± 0.2	9.0 ± 1.3	8.3 ± 0.5	1.3 ± 0.1
48	0.0 ± 0.0	1.2 ± 0.3	0.0 ± 0.0	1.3 ± 0.1	22.7 ± 5.4	1.7 ± 0.6	2.0 ± 0.4	1.0 ± 0.2

MP, methylprednisolone.

**Table 3:** Pharmacokinetic data for MPSS and MP in tissues and plasma in 44 rabbits.

	Plasma		Vitreous		Aqueous humor		Iris		Sclera		Lens		Choroid and retina		Optic nerve	
	MP	MPSS	MP	MPSS	MP	MPSS	MP	MPSS	MP	MPSS	MP	MPSS	MP	MPSS	MP	MPSS
*T *_max_	0.500	0.250	0.500	0.500	4.00	1.00	0.500	0.250	0.500	0.250	6.00	0.500	0.500	0.250	0.500	0.500
AUC_0-*t*_	3730	1936	871	3568	3910	2949	457	825	9846	71530	399	430	1034	1892	708	1913
*t *_1/2_	3.29	13.2	9.49	38.5	1.78	1.90	16.0	56.8	58.2	233	12.7	21.3	27.4	60.1	15.5	49.5
AUMC	10362	1599	3900	15825	16632	10948	2478	6280	35822	210936	6430	5250	6388	16471	2489	6538
*k*	0.211	0.0525	0.0730	0.0180	0.389	0.365	0.0433	0.0122	0.0119	0.00297	0.0546	0.0325	0.0253	0.0115	0.0447	0.014
AUC_0-∞_	3747	1940	887	4379	3954	2950	487	1202	11754	127086	430	492	1113	2501	730	2299
MRT	2.78	0.826	4.48	4.44	4.25	3.71	5.42	7.61	3.64	2.95	16.1	12.2	6.18	8.71	3.52	3.42
*C *_max_	1290	4478	329	3271	643	822	254	776	3960	48050	22.0	252	504	1052	542	1627

MPSS, methylprednisolone sodium succinate; MP, methylprednisolone; *T*_max_, time of peak concentration; AUC, area under the curve, represents exposure; *t*_1/2_, half life time; AUMC, area-under-the-moment-curve; *k*, elimination rate constant; MRT, mean residence time; *C*_max_, maximum concentration.

## Data Availability

No data were used to support this study.

## Abbreviations

MPSS: Methylprednisolone sodium succinate
MP: Methylprednisolone.

## Additional Points

(1) Pharmacokinetics of MPSS after periocular injection was assessed in rabbits. (2) MPSS can be effectively delivered to the posterior segment of the eyes. (3) MPSS is rapidly converted into its active ingredient (MP) in eye tissues. (4) Periocular injection is an effective way to deliver drugs to the eye tissues. (5) The sclera is both a barrier and a reservoir for local release of drugs.

## References

[1] Woods A. C. (1950). Clinical and experimental observation on the use of ACTH and cortisone in ocular inflammatory disease. *American Journal of Ophthalmology*.

[2] Lobo A. M., Sobrin L., Papaliodis G. N. (2010). Drug delivery options for the treatment of ocular inflammation. *Seminars in Ophthalmology*.

[3] Jabs D. A., Rosenbaum J. T., Foster C. S. (2001). Guidelines for the use of immunosuppressive drugs in patients with ocular inflammatory disorders: recommendations of an expert panel. *American Journal of Ophthalmology*.

[4] Jabs D. A. (2004). Treatment of ocular inflammation. *Ocular Immunology and Inflammation*.

[5] Al-Habet S. M., Rogers H. J. (1989). Methylprednisolone pharmacokinetics after intravenous and oral administration. *British Journal of Clinical Pharmacology*.

[6] Jiang S., Chappa A. K., Proksch J. W. (2009). A rapid and sensitive LC/MS/MS assay for the quantitation of brimonidine in ocular fluids and tissues. *Journal of Chromatography B*.

[7] Zammataro A., Civiale C., Saletti R., Foti S. (2011). Development and validation of a liquid chromatography/electrospray ionization tandem mass spectrometry method for the quantification of latanoprost free acid in rabbit aqueous humor and ciliary body. *Journal of Mass Spectrometry*.

[8] Zhang S. Q. (2011). Quantification of triamcinolone acetonide in ocular tissues after intravitreal injection to rabbit using liquid chromatography-tandem mass spectrometry. *Journal of Chromatography B*.

[9] Behar-Cohen F., Gauthier S., Elaouni A. (2001). Methylprednisolone concentrations in the vitreous and the serum after pulse therapy. *Retina*.

[10] Park H. J., Lee J. E., Kim S. I. (2014). Intravitreal pharmacokinetics after posterior subtenon triamcinolone acetonide injection in vitrectomized rabbit eyes. *Retina*.

[11] Ambati J., Canakis C. S., Miller J. W. (2000). Diffusion of high molecular weight compounds through sclera. *Investigative Ophthalmology & Visual Science*.

[12] Ambati J., Gragoudas E. S., Miller J. W. (2000). Transscleral delivery of bioactive protein to the choroid and retina. *Investigative Ophthalmology & Visual Science*.

[13] Maurice D. M., Polgar J. (1977). Diffusion across the sclera. *Experimental Eye Research*.

[14] Rudnick D. E., Noonan J. S., Geroski D. H., Prausnitz M. R., Edelhauser H. F. (1999). The effect of intraocular pressure on human and rabbit scleral permeability. *Investigative Ophthalmology & Visual Science*.

[15] Ranta V. P., Urtti A. (2006). Transscleral drug delivery to the posterior eye: prospects of pharmacokinetic modeling. *Advanced Drug Delivery Reviews*.

